# OWSum: algorithmic odor prediction and insight into structure-odor relationships

**DOI:** 10.1186/s13321-023-00722-y

**Published:** 2023-05-07

**Authors:** Doris Schicker, Satnam Singh, Jessica Freiherr, Andreas T. Grasskamp

**Affiliations:** 1 Sensory Analytics and Technologies, Fraunhofer Institute for Process Engineering and Packaging IVV, Giggenhauser Straße 35, 85354 Freising, Germany; 2 Department of Psychiatry and Psychotherapy, Friedrich-Alexander-Universität Erlangen-Nürnberg, Schwabachanlage 6, 91054 Erlangen, Germany

**Keywords:** Olfaction, Structure-odor relationships, Odor prediction

## Abstract

We derived and implemented a linear classification algorithm for the prediction of a molecule’s odor, called Olfactory Weighted Sum (OWSum). Our approach relies solely on structural patterns of the molecules as features for algorithmic treatment and uses conditional probabilities combined with tf-idf values. In addition to the prediction of molecular odor, OWSum provides insights into properties of the dataset and allows to understand how algorithmic classifications are reached by quantitatively assigning structural patterns to odors. This provides chemists with an intuitive understanding of underlying interactions. To deal with ambiguities of the natural language used to describe odor, we introduced descriptor overlap as a metric for the quantification of semantic overlap between descriptors. Thus, grouping of descriptors and derivation of higher-level descriptors becomes possible. Our approach poses a large leap forward in our capabilities to understand and predict molecular features.

**Supplementary Information:**

The online version contains supplementary material available at 10.1186/s13321-023-00722-y.

## Introduction

The sense of smell is one of the five classical human senses and plays an important role in our everyday life. Even newborns demonstrate olfactory capabilities by responding to the smell of their mother’s breasts [1] and human mate choice could be influenced by odor preferences [2]. Food odors influence appetite and hunger [3, 4] and threat-relevant odors can strengthen human fear memory [5]. Despite their apparent importance, the English language has no abstract words for odors as it has for colors (e.g. “blue”) and even native speakers struggle when naming smells [6]. Our perception of odors, and therefore the naming of smells depends on many subjective factors such as age, cultural background or personal experience [7], or training (compare wine experts [8, 9]). Odors also play a significant economic role, particularly in the food or cosmetic industry, where the development of new aromas and flavors and the identification of odor active molecules is essential. For the creation of new odorants, a predictive approach is necessary during molecular design to reduce the space of candidate molecules from virtually anything to a promising range of molecule structures. Though many advances in odor prediction have been achieved in recent years [10–20], we unfortunately still know little about the relationship between a molecule’s structure and its odor [21–23] to an extent where we can provide chemists with a toolbox for designing molecular structures with a specific odor in mind. However, sophisticated computational methods have led to new insights into these relationships [24–26] and allow prediction whether a molecule is odorous at all [27]. Adding to the hurdles in the field, there is dispute over the dimensionality of the odor space [7, 28]. To derive the rather vague property of odor from objectively measurable or computable molecular properties, a relationship between physicochemical parameters and odor can be employed. As such, the pleasantness of molecules was identified as one of the main dimensions in human olfactory perception [29-31]. Several methods have been proposed to predict the pleasantness of molecules [32] or odor mixtures [33, 34]. Overall, more and more machine learning approaches are applied in human olfactory research [35]. These can be combined with electronic noses [32, 36, 37], GC [38], MS [39, 40], or GC-O methodology [12].

To predict a specific odor, Keller et al. [10] explored the performance of 22 different machine learning models regarding the prediction of 19 odor descriptors. Based on the good performance of linear models, the authors concluded that a linear, additive effect of the features on olfactory perception exists [10]. However, non-linear approaches like random forest and deep neural networks as well achieve high predictive accuracies [11, 15, 16, 41], also for predicting the odor of mixtures [13]. Above-mentioned models use a wide range of computed molecular features and not all of them are easily interpretable. Physicochemical as well as structural features were obtained for example using the Dragon chemoinformatics software (Kode Chemoinformatics, Pisa), PaDel [42], or Mordred [43].

Though promising and useful in their own rights, the models mentioned above use a wide range of different features that do not allow a deep insight into the mechanism of prediction. In addition, due to the opaque nature of the algorithms, the models in previous work rather act as black boxes. Therefore, even if high accuracies are achieved that enable a reliable odor prediction, we still lack knowledge of structure-odor relationships using these models. Further, to predict an odor, the corresponding molecule has to be already synthesized and/or knowledge about physical properties must exist. In addition, though naming smells is subjective, information was rarely provided on why specific descriptors were used for the prediction. As such, clustering odor descriptors is an effective strategy for predicting structure-odor relationships [37, 44], but also the use of word embeddings [45]. Using quasi-primary odors [46] instead of specific descriptors could also reduce the dimensionality of descriptors.

**Fig. 1:**
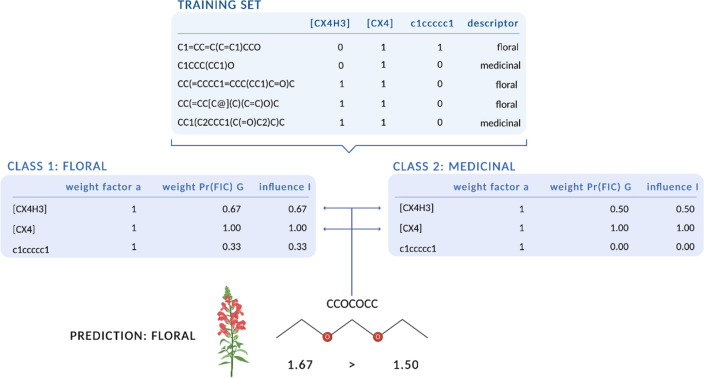
Schematic workflow of a two-dimensional prediction of the odor of a molecule using same-weighted OWSum. A training set contains molecules together with their descriptors (here floral and medicinal) and extracted features that are structural patterns. For simplicity, we only regard three features. Based on the training set, OWSum calculates the influence I by multiplying the weight G with the weighting factor a (here 1 as we use the same-weighted OWSum). For the prediction of a molecule, all features that occur in that molecule are considered, in this case the first and the second feature ([CX4H3] and [CX4]). By summing up their influence, OWSum calculates one score per descriptor. As the score for floral (1.67) is higher than the score for medicinal (1.50), OWSum predicts the odor floral. As floral is in fact the odor of the molecule, the prediction is accepted as correct. See the Methods section for a detailed explanation of the algorithm. (Created with BioRender.com)

In this paper, we present the new linear classification algorithm Olfactory Weighted Sum (OWSum) which is based on conditional probability models and the established algorithm AWSum [47]. OWSum calculates a conditional probability for each feature (i.e. structural pattern) and class (i.e. odor descriptor) that can be further modified by applying a weighting function. This results in an influence value I per feature and descriptor. The highest sum per descriptor of all influence values of occurring features in a molecule predicts the odor (see Methods for a detailed explanation as well as Fig. 1 for a schematic overview).

Besides the prediction of odors, OWSum provides insights into the prediction process and allows ranking structural patterns and identifying their impact on the odor of a molecule. Quality in the choice of descriptors used to label odor impressions is crucial for predictive power. As such, we implemented the metric descriptor overlap, quantifying semantic similarity of two descriptors. OWSum builds on the concept that the overall shape of a molecule is responsible for its odor [48, 49]. As properties like the molecular weight or topological molecular indices are also a result of the chemical structure of a molecule, OWSum succeeded to use solely the structure and its patterns (chemical fragments) as features for prediction. As a direct consequence, this approach gives insight into the relationship between a molecule’s structure and its odor. OWSum quantifies this relationship by assigning each structural pattern a value for its influence on an odor percept. This value can be interpreted as the impact of the pattern for the odor.

To analyze the semantic overlap of descriptors using descriptor overlap and perform odor prediction as well as gaining insight into structure-odor relations using OWSum, we used molecules and their odors from Dravnieks’ database [50]. Our explicit databases are described in detail in the method section. Figure 1 shows a schematic overview of the workflow of OWSum.

## Results and discussion

Olfactory databases are often the results of a panel testing, in which panelists provide different descriptors while referring to the same smell due to subjective, individual preferences and experiences [7, 9]. This means that the databases provide a wide range of not necessarily mutually exclusive descriptors, or even describe identical features. A problem with such databases is that for the prediction of the odor of molecules, descriptors should be as selective and specific as possible. Otherwise, the algorithm cannot learn efficiently from the training set. Further, if descriptors are included that describe a wide range of smells, pure guessing on these descriptors would reach high accuracy.

**Fig. 2:**
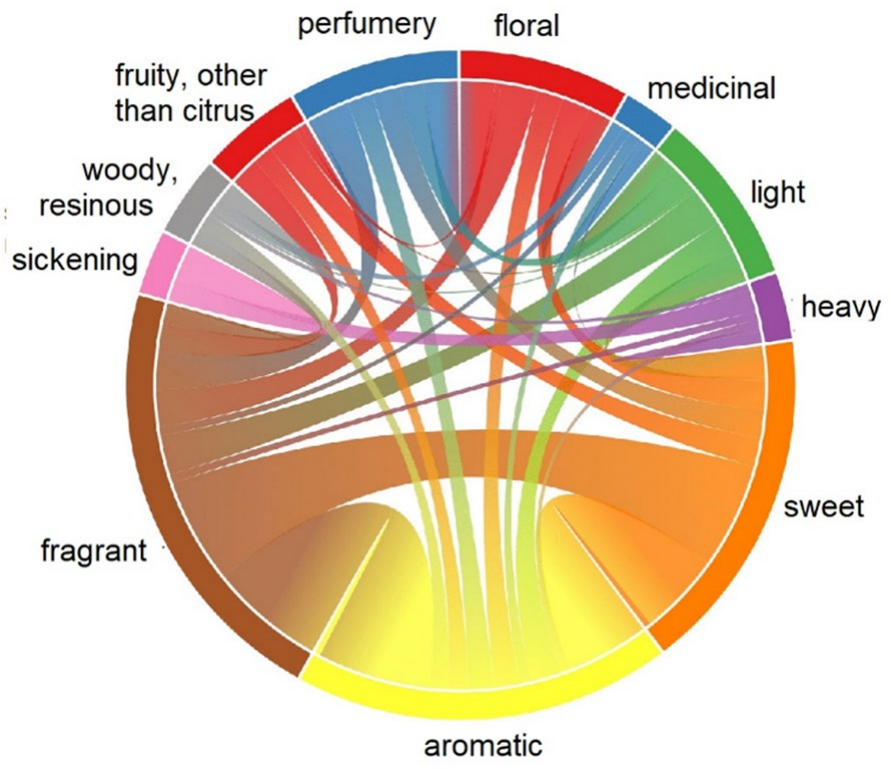
Chord diagram displaying the connections between the eleven descriptors of the database. The thickness of a connection is proportional to the number of molecules belonging to both descriptors. The semi-elliptical area that is only about its starting descriptor is proportional to the total number of molecules smelling like that descriptor. Therefore, comparing the width of this area and the arc allows us to estimate the number of molecules smelling only like that descriptor. Most of the molecules of the upper half have connections to the descriptors ‘fragrant’, ‘aromatic’ and ‘sweet’ that are displayed in the lower half

The metric descriptor overlap allows to optimize the choice of descriptors for prediction and learn about structure-odor relationships. Analyzing 97 odor molecules belonging to eleven olfactory descriptors (‘aromatic’, ‘floral’, ‘fragrant’, ‘heavy’, ‘light’, ‘medicinal’, ‘sickening’, ‘sweet’, ‘woody, resinous’, ‘fruity, other than citrus’ and ‘perfumery’) of Dravnieks’ atlas of odor character profiles [50] (see Method section for details how this database was created) revealed that more than a third of the molecules of the database smell like more than one of these. Figure 2 visualizes this relationship and semantic overlap.

**Fig. 3:**
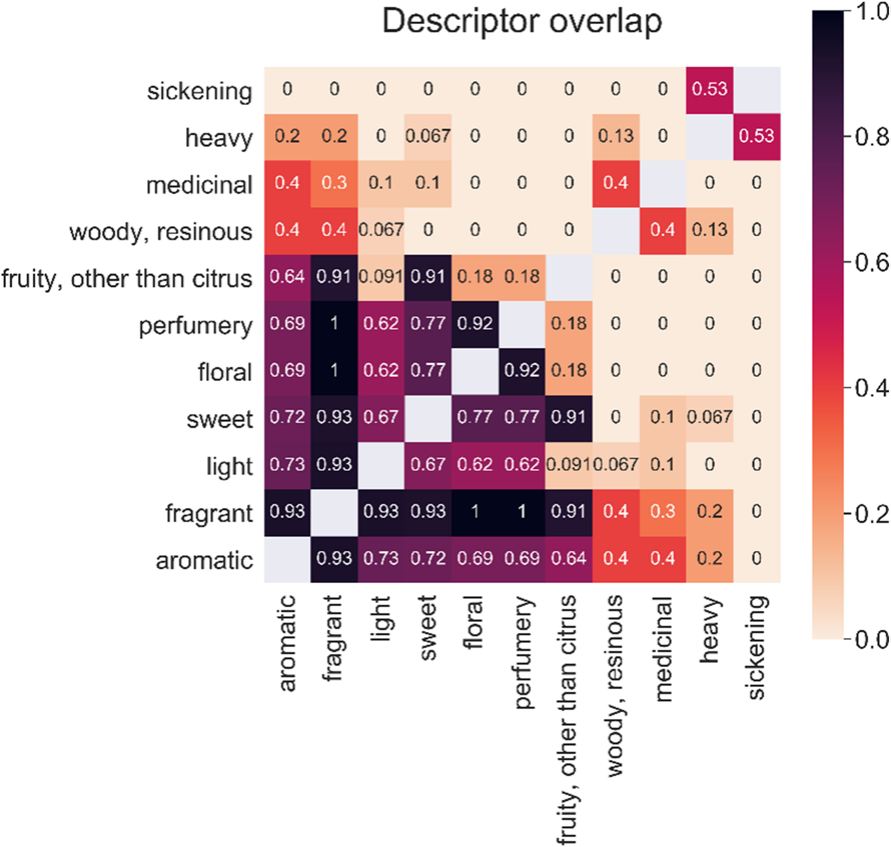
Heat map of the descriptor overlap. A dark color represents a high descriptor overlap and therefore a high analogy between the uses of these descriptors for one smell

Computing the descriptor overlap pairwise for all descriptors, we quantitatively analyzed the descriptors of the database to identify highly similar odors (Fig. 3). ‘Aromatic’ and ‘fragrant’ had a mean descriptor overlap with the other descriptors of over 50%, ‘fragrant’ with the majority of the other descriptors even over 90%. Only in combination with the descriptors ‘heavy’ and ‘sickening’ low descriptor overlaps occurred (< 20%): Whereas ‘aromatic’ and ‘fragrant’ belong to pleasant odors, ‘heavy’ and ‘sickening’ are perceived as unpleasant [51]. This confirms that both ‘aromatic’ and ‘fragrant’ are rather broad-spectrum descriptors and do not describe specific odors [52], but are used for a wide range of different pleasant smells. As such, the two descriptors act as higher-level categories. The same is valid for the descriptor ‘sweet’. After dropping the three descriptors ‘aromatic’, ‘fragrant’, and ‘sweet’, the mean descriptor overlap for all remaining descriptors was lower than 25%. These properties describe specific smells with limited relationships. As a conclusion, the metric descriptor overlap can reliably detect non-selective and non-sensitive descriptors in our database and describe relations in a quantitative way. This can be used to optimize the choice of descriptors for prediction.

### Olfactory prediction performance of OWSum

To predict the specific odor of a molecule, we derived and implemented the novel linear classification algorithm OWSum. OWSum relies solely on structural patterns of the molecules as features for algorithmic treatment (see Fig. 1 for a schematic overview of the workflow of OWSum). We tested several variations of OWSum using five-fold cross-validation, which differ according to the weighting or application of feature selection, to select the best performing one and compare it to multilabel k-nearest neighbors classifier (mlKNN, optimized k = 1) (see Table 1). To be comparable to OWSum, mlKNN was modified to predict the class(es) with the maximum probability per molecule instead of using a threshold. Our dataset consisted of 64 molecules belonging to the descriptors ‘floral’, ‘medicinal’, ‘woody, resinous’, ‘sickening’, ‘fruity, other than citrus’ and ‘perfumery’ that we derived from analyzing Dravnieks’ database [50] with the descriptor overlap (see Method section).

**Table 1:** Performance of OWSum and mlKNN (optimized k = 1) regarding the prediction of the descriptors ‘floral’, ‘medicinal’, ‘woody, resinous’, ‘sickening’, ‘fruity, other than citrus’ and ‘perfumery’ using five-fold cross-validation. One-versus-rest ROC AUC values and MCC values are the averaged results over all classes. See Supplementary Material for ROC AUC and MCC values per class as well as ROC curves per odor for the best-performing variant

Feature selection	Weighting factor a_i,j_ for OWSum or mlKNN	Overall accuracy (%)^a^	Predicted accuracy (%)^a^	Non- predictable molecules (%)	Mean ROC AUC (underestimated)^a^	Mean ROC AUC (overestimated)^a^	Mean MCC (underestimated)^a^	Mean MCC (overestimated)^a^
		Five-fold cross-validation						
-	Same- weighted	46.8	46.8	0	0.62	0.67	0.24	0.40
idf	Same-weighted	56.3	58.1	3.2	0.66	0.77	0.32	0.47
idf	Tf-idf-weighted	75.0	77.6	3.2	0.75	0.81	0.47	0.63
idf	Tf-idf-weighted ∙ 1/Pr(F|C)^b^	68.8	71.4	3.2	0.71	0.76	0.41	0.57
idf	Tf-idf-weighted ∙ 1/Pr(F|C) Pr(C|F)^b^	64.1	66.6	3.2	0.70	0.75	0.38	0.54
-	mlKNN	69.0	69.0	0	0.77	0.82	0.53	0.62
idf	mlKNN	61.2	61.2	0	0.74	0.79	0.48	0.55

^a^Defined in the Methods Section

^b^We divide by the weight Pr(F_j_|C_i_) in order to find the importance of this weight and compare the improvement using Pr(F_j_|C_i_) and not Pr(C_i_|F_j_) as AWSum does [47]

All variations of OWSum were more than twice as performant as expected from random guessing. Pure guessing on one of the six descriptors would achieve an accuracy of 21.4% (on average, a molecule smells like 1.28 different descriptors). Additionally, OWSum outperformed mlKNN in terms of accuracy. OWSum in combination with tf-idf-weighting performed best with a predicted accuracy of 77.6%. This means that using the conditional probability that a structural pattern belongs to a molecule under the condition that the molecule smells like an odor multiplied with the tf-idf value for this structural pattern was the best method to calculate the influence of the structural pattern for the odor. In all cases where we applied feature selection using idf values or tf-idf-weighting, OWSum could not predict 3.2% of the molecules and therefore the overall accuracy was lower than the predicted accuracy. These 3.2% were the two molecules hexanol (C_6_H_14_O, descriptor: ‘woody, resinous’) and thiophene (C_4_H_4_S, descriptors: ‘sickening’). We shortly describe the reasons for that: Hexanol does not have any features that do not occur in each descriptor class. That means, all the features are extremely unspecific with an idf value equal to zero. Thiophene, on the other hand, in addition to such unspecific ones, exhibits features that are unique to thiophene in our database. That means that OWSum has not trained on these features due to five-fold cross-validation and can therefore not consider them. In all cases where OWSum made a prediction, OWSum only predicted one descriptor. The prediction was therefore precise. Using OWSum with tf-idf weights, we achieved a training accuracy (i.e. we trained and tested on the whole dataset) of 90.5%. Out of all the 64 molecules, only the molecule hexanol was not predictable. This evaluation of the model showed that OWSum can replicate the odor of molecules by splitting them into their structural patterns. This also suggests that by using larger datasets, the performance of OWSum can be improved even further. In addition, if we want to leverage OWSum to gain insights about structure-odor relationships instead of predicting molecules, the usage of OWSum on the complete dataset is an accurate approach (see next section).

### Structure-odor relationships

Apart from solving classification problems, in particular odor prediction, OWSum also allows gaining insight into the classification. According to the high accuracies when predicting the odor of molecules, the principle of using structural patterns and their relevance to predict the odor is a good approach. More precisely, the influence I_i,j_ is a value that quantifies the impact of a structural pattern i on the odor j of a molecule. This value is optimized if we use the conditional probability that a structural pattern occurs in a molecule given the condition that this molecule smells like a specific odor and multiply it with the tf-idf value of the pattern (see above section). Applying OWSum, we can extract these influences and gain direct insight into the prediction. This also allows us to learn about structure-odor relationships. For this aim, we trained OWSum on all of the available molecules.

We first looked at the number of features that can be extracted for molecules of a given odor and the number of features that remain after dropping the ones with an idf value equal to zero (Additional file 1: Figure S2). Those features are assigned a weight of zero for all descriptor classes and are thus not important for the classification process. Features that were dropped because of an idf value equal to zero are especially small structural patterns that occur in nearly all molecules like [CX4]. There was a high variability in the number of extracted features per descriptors. This was independent of the number of molecules per descriptor: For example, fewer than 5000 features with an influence greater than zero belonged to the descriptor ‘sickening’ with 20 molecules, whereas more than 53,000 features belonged to the descriptor ‘woody, resinous’ with only 15 molecules. As a conclusion, molecules smelling like ‘sickening’ are more similar and probably less complex than molecules smelling like ‘woody, resinous’.

To get deeper insight which features had a high impact on the odor of a molecule, we extracted the features with the highest influence per descriptor (Table 2). Molecules smelling like ‘perfumery’ and ‘floral’ had the exact same most important 57 features. This is in line with the high descriptor overlap for these descriptors of 92%, suggesting to combine these groups of molecules. All these features contained a double bond between two carbon atoms, making this the most important characteristic. Outstanding structural patterns for molecules smelling like ‘woody, resinous’ were branched alkyls. ‘Woody’ odorants are associated with rigid bulky hydrocarbon skeletons [23]. Whereas a subgroup discovery algorithm revealed the rule that ‘woody’ molecules are hydrophobic and rather not cyclic nor aromatic [24], investigations using a Transformer model suggested that woody molecules are often ring structures [26]. This is in accordance with our results, where cyclohexane structures were assigned the second highest influence values whereas aromatic structures scored low. Oxygen atoms had high impact for the descriptors ‘medicinal’, ‘sickening’ and ‘fruity, other than citrus’. For the latter, the oxygen occurred as an ester. We cannot make such a specification for molecules belonging to ‘sickening’ as the oxygen occurred as an ester or acid. For ‘medicinal’, the oxygen was single-bonded to the chain.

**Table 2:** Important features per descriptor. For each descriptor, we give the value of the highest influence and the number of features with this influence. We present the smallest of these features and another example

Descriptor	Floral	Perfumery	Woody, resinous	Medicinal	Sickening	Fruity, other than citrus
Highest influence value [× 10^–6^]	17	14	13	77	354	110
Number of features with this influence	57	57	101	124	1	13
Smallest characteristic feature						
SMARTS of this feature	[#6](-[#6])(-[#6]) = [#6]	[#6](-[#6])(-[#6]) = [#6]	[#6](-[#6](-[#6]-[#6])-[#6]-[#6])-[#6]	[#6]:[#6]-[#8]	[OX2H1][CX3](= [OX1])[#6]	[#6](-[#6](-[#8]-[# 6]-[#6]) = [#8])-[#6]
Smallest feature with another characteristic for this influence or second example					-	
SMARTS of this feature	[#6]-[#6] = [#6]-[#6]	[#6](-[#6]) = [#6]-[#6]	[#6](-[#6])-[#6](-[#6]-[#6]-[#6]-[#6])-[#6](-[#6])-[#6]	[#6]:1(:[#6]:[#6]:[#6]:[#6]:[#6]:1)-[#8]	-	[#8] = [#6](-[#6]-[#6])-[#8]-[#6]-[#6]

**Fig. 4:**
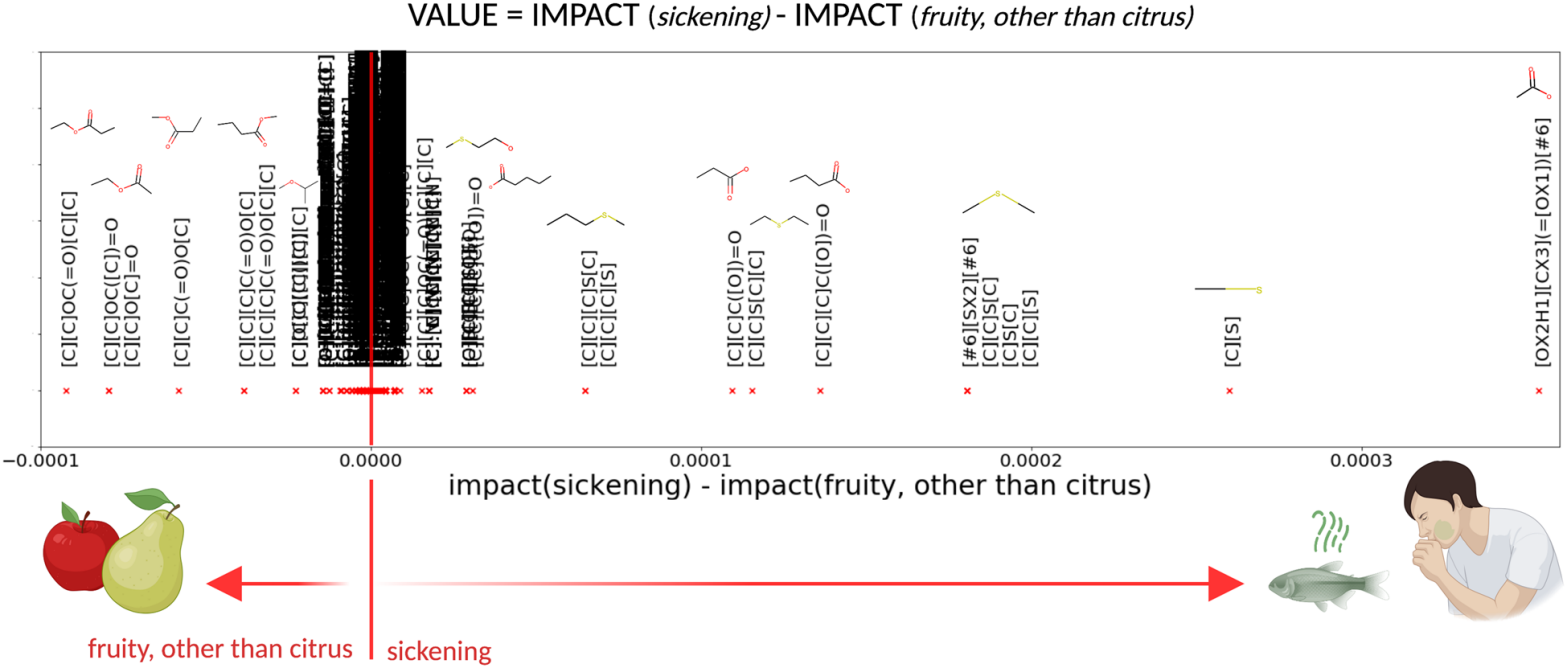
Importance of features for ‘fruity, other than citrus’ vs. ‘sickening’. By applying OWSum on molecules of the six descriptors ‘floral’, ‘medicinal’, ‘woody, resinous’, ‘fruity, other than citrus’ and ‘perfumery’, we can extract quantitative values for structural patterns per descriptor. In this image, we display the difference between the influence value for features for the descriptors ‘fruity, other than citrus’ and ‘sickening’. To better visualize the important patterns, we combined all SMARTS-patterns if they belonged to a SMILES-structure in case they stand alone and display these.  (Created with BioRender.com)

Above-mentioned features had a high impact on one odor of a molecule, but there is no guarantee that the molecule smells like this odor. OWSum considers this, as the algorithm uses every single pattern of a molecule to classify it. With OWSum, we can rank the features for each descriptor. As this is hard to display in a six-dimensional case, we show this for features of the two descriptors ‘fruity, other than citrus’ and ‘sickening’ in Fig. 4.

We cannot make general assumptions regarding what a molecule will smell like if it has a special feature in it. We can only assume e.g., that having a sulfur atom increases the probability that the molecule smells like ‘sickening’, in accordance with literature stating that sulphurous molecules are perceived as unpleasant [23] or decayed [53]. We cannot make such assumptions regarding esters. Using OWSum, the sole occurrence of an ester had no impact, as the feature was not included in the classification because of an idf-value equal to zero. That means this structural pattern was too general. If the ester occurred in a specific combination with other features, though, it was specific enough to be a predictor. The occurrence of an ester with at least two carbon atoms on each side was a feature with the highest influence for molecules smelling like ‘fruity, other than citrus’ (see Table 2). This confirms that the main group of fruity odorants are esters [23], but esters are neither a necessary nor a sufficient criterion for a molecule to smell fruity [21, 23].

To summarize, odor prediction needs to include a wide range of structural patterns. These patterns have a summative influence on odor perception. OWSum not only considers this for prediction but also quantifies relationships by assigning each structural pattern a value for its influence on an odor percept.

## Conclusions

We developed the linear classification algorithm OWSum that uses the statistical methods conditional probability and tf-idf function which is often used in text retrieval systems. Our algorithm allows gaining insight into the process of arriving at a specific decision. By changing the weighting factor, the algorithm can be easily adapted to different classification problems and improved for better accuracies. In addition to the algorithm, we introduced the new metric descriptor overlap. Using this, we can quantify the semantic overlap between several odor descriptors. This allows grouping or detecting higher-level descriptors. We applied OWSum on molecules and used solely their structural patterns as features to predict their odor. As such, OWSum allows olfactory prediction even before synthesizing new molecules and without knowledge about physical properties in contrast to previously proposed methods. Further, the workflow of OWSum is easily understandable and comprehensible. Therefore, OWSum does not only make reliable predictions but also allows us to infer knowledge about structure-odor relationships as quantitative values are assigned to structural patterns that describe the impact of the patterns for different odors. Using these values, further analysis about structure-odor relationships can be accomplished in the future. Moreover, our proposed algorithm is applicable to other classification problems, including the prediction of other molecular properties such as toxicity, and poses a large leap forward in our capabilities to understand underlying structural reasons.

## Methods

The algorithm described here serves to use structural features of molecules to infer their most relevant odor quality. A set of statistical methods was used, including the validation of our results, as described in the following section.

### The classification algorithm OWSum

The algorithm OWSum was structured into three steps:

1. Feature selection
2. Calculation of the influence of the features regarding the classes
3. Classification

**Fig. 5:**
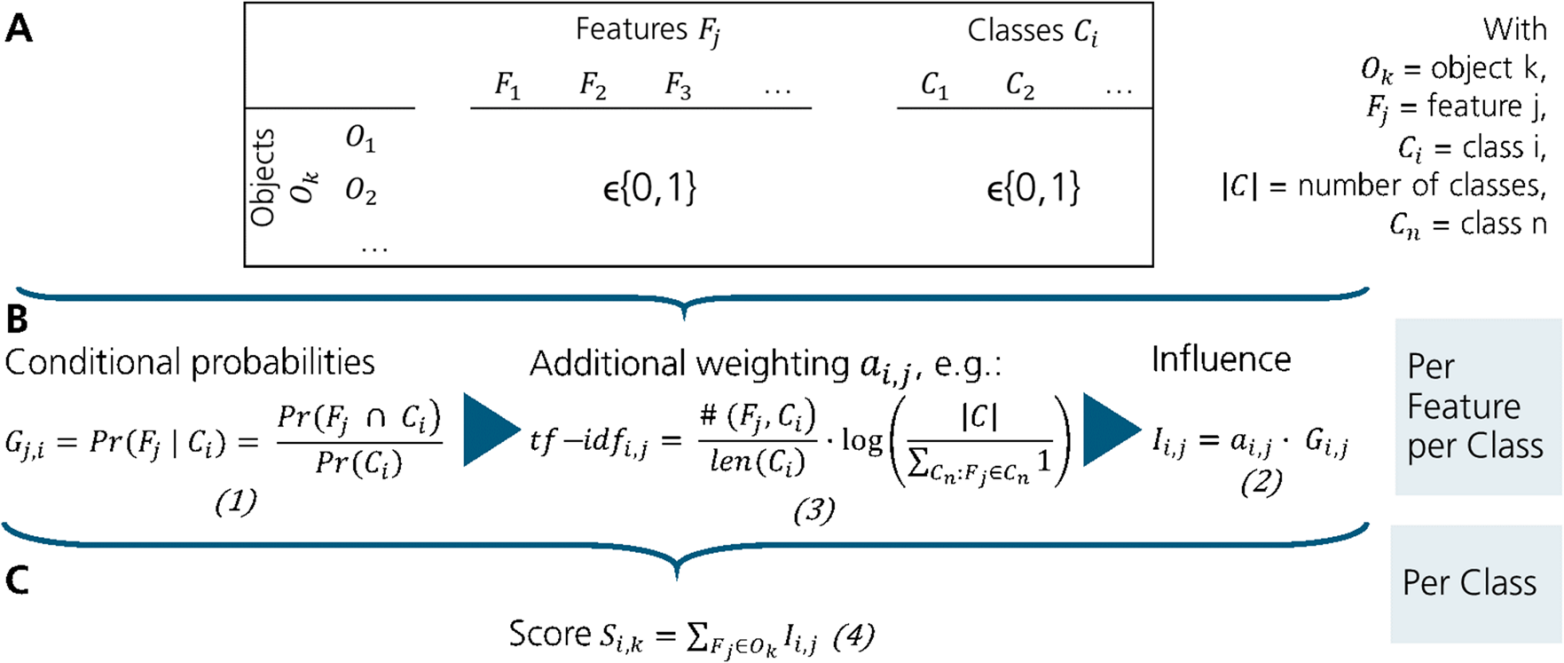
Overview of the classification algorithm OWSum. **A** Input matrix: Objects O_k_ can have different features F_j_ and belong to one or more classes C_i_. If a feature occurs to an object or if the object belongs to a class, the values of the cells are 1 otherwise 0. **B** Calculation of the influence values using Eqs. 1 and 2 and the tf-idf value (Eq. 3) as weighting factor (compare step 2) **C** Calculation of the score the prediction is based on (Eq. 4, compare step 3)

To make the description of the algorithm OWSum clear, we describe steps 2 and 3 before the feature selection. As OWSum can not only be applied for odor prediction but for many classification problems, we first describe the algorithm in general before specifying it for odor prediction. To familiarize the reader with used variables, indices and equations, a schematic overview is displayed in Fig. 5. An applied schematic overview for odor-prediction with a simplified example is shown in Fig. 1.

**Step 2: Calculation of the influence of the features regarding the classes.** OWSum was based on the idea that each feature of the objects has a special influence on a class. To calculate this influence of a feature, we used the conditional probability of the feature value given the class. More specifically, for a feature ${F}_{j}$ and a class ${C}_{i}$, the probability that ${F}_{j}$ occurred under condition ${C}_{i}$, was calculated by $Pr\left({F}_{j}|{C}_{i}\right)$ and was called the weight ${G}_{j,i}$ (Eq. 1).

1 $$G_{{j,i}} = Pr(F_{j} {\text{~}}|{\text{~}}C_{i} ) = {\text{~}}\frac{{Pr\left( {F_{j} {\text{~}} \cap {\text{~}}C_{i} } \right)}}{{Pr\left( {C_{i} } \right)}}$$

$$G_{j,i} = {\text{ weight of the jth}} {\text{feature for class i}}$$

$$F_{j} = {\text{feature j}}$$

$$C_{i} = {\text{class i}}$$

To add additional information and therefore improve the performance of OWSum, a weighting factor ${a}_{i,j}$ could be multiplied to the weight ${G}_{j,i}$. This gave a single value called influence ${I}_{i,j}$ of a feature ${F}_{j}$ for a special class ${C}_{i}$ (Eq. 2). If the same-weighted OWSum was used, all weighting factors ${a}_{i,j}$ were set to 1.

2 $$I_{{i,j}} = a_{{i,j}} \cdot G_{{i,j}}$$

$$I_{{i,j}} = {\text{ influence of the jth feature for class i}}$$

$$a_{{i,j}} = {\text{ weight of the jth feature for class i}}$$

$$G_{{j,i}} = {\text{ weight of the jth feature for class i}}$$

Another approach was to consider the relevance of a feature as a weighting factor on the classification. Therefore, we could use the tf-idf value that is mostly used for information retrieval systems and document formalization [54]. The tf-idf value is the multiplication of the term frequency tf and the inverse document frequency idf (Eq. 3). Using the tf value, features with higher frequency were weighted as more important than features with a lower frequency. The idf value considered that a feature was more important if it was specific and not distributed over many classes.

3 $$tf - idf_{{i,j}} = \frac{{\# (F_{j} ,C_{i} )}}{{len(C_{i} )}} \cdot {\text{log}}\left( {\frac{{|C|}}{{\sum\limits_{{C_{n} :F_{j} \in C_{n} }} 1 }}} \right)$$

$${F}_{j}$$ = feature j

$${C}_{i}$$= class i

$\left|C\right|$ = number of classes

$${C}_{n}$$= class n

If we used the tf-idf values as weighting factors, we call this variation of the algorithm the tf-idf-weighted OWSum.

**Step 3: Classification.** To predict the class of an object, we had to consider all features occurring in that object. Therefore, for all features of that object O_k_, all influences ${I}_{i,j}$ for a class ${C}_{i}$ were added to a score ${S}_{i,k}$ (Eq. 4).

4 $$S_{{i,k}} = \sum {F_{j} \in O_{k} } I_{{i,j}}$$

${S}_{i,k}$= score for the k th object to belong to class i

$${F}_{j}$$= feature j

$${O}_{k}$$ = object k

${I}_{i,j}$ = influence of the j th feature for class i.

As a result, for an n-dimensional classification problem we got n scores. OWSum made the prediction by selecting the class(es) with the highest score. If an object belonged to more than one class and OWSum correctly predicted a subset of these, the prediction was accepted as correct. If OWSum predicted all possible classes, we considered this object as unpredictable as no valuable prediction could be given. Therefore, we did not only have true and false predictions but non-predictable objects as well.

**Step 1: Feature selection.** We could improve the accuracy of OWSum by applying feature selection as a first step. For our case, we used feature selection based on idf values (compare Eq. 3, second factor). This dropped all the features that had an idf value equal to zero. These features occurred in all classes and were therefore not specific enough to contribute to the prediction.

### Validation of OWSum

For the validation of the algorithm, we calculated accuracies, ROC AUC, and MCC values with five-fold cross-validation.

Accuracies: The overall accuracy was the proportion of correct predictions among the total number of examined cases (Eq. 5). We also calculated the predicted accuracy, which was the proportion of correct predictions among the total number of cases where OWSum made a prediction (Eq. 6). The predicted accuracy is a better approach if we were interested in how many molecules had been predicted incorrectly.

5 $$predicted\,accuracy= \frac{\# true\,positives}{\# all\,predictable\,molecules}$$

6 $$overall\,accuracy=\frac{\# true\,positives}{\# all\,tested\,molecules}$$

ROC AUC and MCC: As there can be multiple descriptors per object but OWSum only predicts one descriptor, we calculated two versions of one-versus-rest ROC AUC values: If a molecule has more than one descriptor (e.g. molecule A smells perfumery and floral) and one of those was predicted (e.g. floral), the ROC AUC value for this descriptor (floral, label 1) against rest (label 0) is calculated using a true label of 1 and a predicted label of 1. If, however, the ROC AUC for another of those descriptors (e.g. perfumery) is calculated, a true label of 1 and a predicted label of 0 is used (“underestimated ROC AUC” that is a lower bound). In this case, the prediction is treated as a false prediction – even if OWSum predicted another correct descriptor. Thus, resulting ROC AUC values underestimate the predictive performance of OWSum. An alternative is to assign a true label of 0 (rest class) to the molecule (this is also correct, as at least one descriptor is in the rest class), the predicted value is again 0 (“overestimated ROC AUC” that is an upper bound). This however overestimates the predictive performance for this specific descriptor. We provide ROC curves per descriptor for the best-performing variant of OWSum in the Additional file 1. Analogous to above, we calculated under- and overestimated one-versus-rest MCC values. If the prediction vector and the ground truth just consisted of zeros, we used the strategy defined in [55], where in such a situation, the MCC is set as 1, providing us with an upper bound value for this metric. This only occurred for the overestimated MCC values.

### OWSum for odor prediction

When using OWSum as a classification algorithm to predict the odor of molecules, a class represents an olfactory descriptor, objects refer to the molecules and features are structural patterns within these molecules. These structural patterns were extracted from the chemical structure of the molecules. The molecules were encoded as Simplified Molecular Input Line Entry Specification (SMILES) [56, 57] and features were encoded as SMILES ARbitrary Target Specification (SMARTS) [58]. As the features were not exclusive but organized in a hierarchical structure, the number of occurrences of a feature was stored indirectly through the occurrence of another, higher feature in that molecule (e.g. that has a longer chain or additional elements). OWSum only considered whether a feature occurred in a molecule, not its frequency. This prevented an overrepresentation of small features. Figure 1 shows the workflow of OWSum using a simplified example. To test the performance and robustness of OWSum, we used five-fold cross-validation.

### The metric descriptor overlap

To quantify the overlap of two descriptors, we introduced the new metric descriptor overlap. The descriptor overlap is the proportion of the number of molecules described by both descriptors to the number of molecules of the rarer descriptor (Eq. 7). An example is given in Additional file 1: Figure S3.

$$descriptor\,overlap \left(desc1, desc2\right)= \frac{\#{M}_{desc1\,\cap desc2} }{\underset{\mathit{desc} \in \mathit{desc}1, \mathit{desc}2}{\mathrm{min}}(\# {M}_{desc})}$$

7 $$descriptor\,overlap \left(desc1, desc2\right) \in \left[0, 1\right]$$

$${\text{desc1}},{\text{ desc2 }} = {\text{ descriptors}}$$

$${\text{M}}_{{\text{x}}} = {\text{ object with descriptor x}}$$

In terms of molecules as objects and olfactory descriptors, a high descriptor overlap is an indication that the two descriptors refer to the same odor or one of the descriptors is a more general one that includes the other descriptor. If the descriptor overlap between two descriptors desc1 and desc2 is equal to one and desc1 has more molecules than desc2, all molecules smelling like desc2 also smell like desc1. A descriptor overlap of zero would mean that no molecule smells like both of the descriptors.

### Database

For odor prediction and gaining insight using OWSum and the descriptor overlap, we used molecules and their descriptors from Dravnieks’ atlas of odor character profiles [50]. For this purpose, we binarized the features of the molecules in the dataset by first calculating the maximum common substructure between each pair of molecules in a reference corpus of molecules to create a reference dataset of features. The reference corpus for our task was the ZINC dataset [59] with all molecules under molecular weight of 200 Da and as a further filter, only molecules marked as ‘in-stock’ were selected. In total, there were 263,921 molecules in the reference dataset.

As we were interested in the characteristic and most-perceived odors of a molecule, we only assigned a descriptor to a molecule if its percentage of applicability was at least 25%. Furthermore, we only considered descriptors that matched at least ten molecules. This was important to have enough data for the training and testing of OWSum. 97 odor molecules and eleven descriptors (‘aromatic’, ‘floral’, ‘fragrant’, ‘heavy’, ‘light’, ‘medicinal’, ‘sickening’, ‘sweet’, ‘woody, resinous’, ‘fruity, other than citrus’ and ‘perfumery’) remained after this initial filtering step. For odor prediction, we excluded descriptors with a mean descriptor overlap over 49%, suggesting a non-selective and non-specific odor representation. To further optimize the choice of descriptors, we dropped ‘heavy’ and ‘light’: According to Iatropoulus et al. [52], a high inconsistency exists between individuals using these descriptors and they are generally not associated with smell [52]. 64 molecules and six descriptors (‘floral’, ‘medicinal’, ‘sickening’, ‘woody, resinous’, ‘fruity, other than citrus’, and ‘perfumery’) remained. On average, a molecule smelled like 1.28 different descriptors, indicating a successful extraction of unique olfactory descriptors. Our final resulting dataset consisted of 64 molecules and their corresponding structural features, which we used as input for OWSum.

### Implementation

In terms of programming, OWSum and the descriptor overlap were implemented in Python 3.7. To determine the performance of OWSum, we performed five-fold cross-validation. Therefore, as well as for calculating ROC AUC and MCC values we used scikit-learn 0.24.1 [60]. To compare OWSum against a multilabel k-nearest neighbors classifier (mlKNN), we used multilabel k Nearest Neighbors from scikit-multilearn 0.2.0 [61]. We optimized k between 1 and 10 using GridSearchCV from scikit-learn. We adapted mlKNN, so that it predicts the class(es) with the maximum probability instead of using a threshold.

## Supplementary Information

**Additional file 1: Table S1**. ROC AUC values per odor. **Table S2**. MCC values per odor. **Figure S1**. ROC Curves for tf-idf-weighted OWSum with idf-feature selection per odor. **Figure S2**. Additional image with descriptive information of our database and extracted features. **Figure S3**. Example for the calculation of the descriptor overlap

## Data Availability

Our used dataset for training and testing as well as the code are available in an OSF repository (https://osf.io/kjh7t/). We used Dravnieks’ Atlas of odor character profiles [50] as original database, available e.g. at https://github.com/pyrfume/pyrfume-data/tree/main/dravnieks_1985. The algorithm as well as all other further preprocessing steps are described in detail in the Method section.
