# Supplementary material for: OWSum: algorithmic odor prediction and insight into structure-odor relationships
Source: J Cheminform. 2023 May 7;15:51. doi: 10.1186/s13321-023-00722-y (PMC10164323; doi:10.1186/s13321-023-00722-y)

OWSum: algorithmic odor prediction and insight into structure-odor relationships

Doris Schicker^a,b^, Satnam Singh^a,b^, Jessica Freiherr^a,b^, and Andreas T. Grasskamp^a^

^a^Sensory Analytics and Technologies, Fraunhofer Institute for Process Engineering and Packaging IVV, Giggenhauser Straße 35, 85354 Freising, Germany

^b^Department of Psychiatry and Psychotherapy, Friedrich-Alexander-Universität Erlangen-Nürnberg, Schwabachanlage 6, 91054 Erlangen, Germany

Corresponding authors: Andreas Grasskamp ([andreas.grasskamp@ivv.fraunhofer.de](mailto:andreas.grasskamp@ivv.fraunhofer.de)) & Doris Schicker ([doris.schicker@ivv.fraunhofer.de](mailto:doris.schicker@ivv.fraunhofer.de))

- Supplementary Material -

**Table S1. ROC AUC values (underestimated / overestimated).**

| Feature selection | Weighting  factor ai,j for OWSum or mlKNN | Floral | Medicinal | Woody,  resinous | Sickening | Fruity, other  than citrus | Perfumery | Mean over  all classes |
| --- | --- | --- | --- | --- | --- | --- | --- | --- |
|  | 5-fold cross-validation | | | | | | | |
| - | Same-weighted | 0.49 | 0.57 | 0.58 | 0.72 | 0.70 | 0.65 | 0.62 |
|  |  | 0.5 | 0.64 | 0.58 | 0.76 | 0.70 | 0.81 | 0.67 |
| idf | Same-weighted | 0.54 | 0.62 | 0.66 | 0.75 | 0.74 | 0.62 | 0.66 |
|  |  | 0.96 | 0.67 | 0.66 | 0.79 | 0.74 | 0.82 | 0.77 |
| **idf** | **Tf-idf-weighted** | **0.74** | **0.84** | **0.90** | **0.74** | **0.84** | **0.47** | **0.75** |
|  |  | **0.96** | **0.86** | **0.90** | **0.78** | **0.84** | **0.50** | **0.81** |
| idf | Tf-idf-weighted ∙  1/Pr(F\|C) | 0.74 | 0.89 | 0.86 | 0.75 | 0.56 | 0.47 | 0.71 |
|  |  | 0.96 | 0.91 | 0.86 | 0.79 | 0.56 | 0.5 | 0.76 |
| idf | Tf-idf-weighted ∙  1/Pr(F\|C)∙ Pr(C\|F) | 0.74 | 0.77 | 0.83 | 0.75 | 0.63 | 0.45 | 0.70 |
|  |  | 0.96 | 0.82 | 0.83 | 0.78 | 0.63 | 0.46 | 0.75 |
| - | mlKNN | 0.75 | 0.80 | 0.85 | 0.84 | 0.60 | 0.76 | 0.77 |
|  |  | 0.86 | 0.82 | 0.85 | 0.90 | 0.64 | 0.87 | 0.82 |
| idf | mlKNN | 0.79 | 0.75 | 0.78 | 0.76 | 0.58 | 0.77 | 0.74 |
|  |  | 0.87 | 0.77 | 0.78 | 0.79 | 0.62 | 0.88 | 0.79 |

Performance of OWSum and mlKNN (optimized k=1) regarding the prediction of the descriptors ‘floral’, ‘medicinal’, ‘woody, resinous’, ‘sickening’, ‘fruity, other than citrus’ and ‘perfumery’ using five-fold cross-validation. One-versus-rest ROC AUC values are provided per class as well as averaged results over all classes. The first line shows underestimated (lower bound), the second line overestimated (upper bound) values per variant. See the Method Section in the main manuscript for a detailed explanation of these metrics.

**Table S2. MCC values (underestimated / overestimated).**

| Feature selection | Weighting  factor ai,j for OWSum or mlKNN | Floral | Medicinal | Woody,  resinous | Sickening | Fruity, other  than citrus | Perfumery | Mean over  all classes |
| --- | --- | --- | --- | --- | --- | --- | --- | --- |
|  | 5-fold cross-validation | | | | | | | |
| - | Same-weighted | -0.03 | 0.21 | 0.27 | 0.41 | 0.29 | 0.31 | 0.24 |
|  |  | 0.60 | 0.32 | 0.27 | 0.47 | 0.29 | 0.47 | 0.40 |
| idf | Same-weighted | 0.08 | 0.31 | 0.46 | 0.42 | 0.36 | 0.27 | 0.32 |
|  |  | 0.67 | 0.38 | 0.46 | 0.47 | 0.36 | 0.46 | 0.47 |
| **idf** | **Tf-idf-weighted** | **0.49** | **0.61** | **0.80** | **0.47** | **0.55** | **-0.06** | **0.47** |
|  |  | **0.85** | **0.65** | **0.80** | **0.54** | **0.55** | **0.40** | **0.63** |
| idf | Tf-idf-weighted ∙  1/Pr(F\|C) | 0.49 | 0.70 | 0.68 | 0.52 | 0.13 | -0.06 | 0.41 |
|  |  | 0.85 | 0.73 | 0.68 | 0.59 | 0.13 | 0.40 | 0.57 |
| idf | Tf-idf-weighted ∙  1/Pr(F\|C)∙ Pr(C\|F) | 0.49 | 0.54 | 0.64 | 0.48 | 0.24 | -0.10 | 0.38 |
|  |  | 0.85 | 0.61 | 0.64 | 0.52 | 0.24 | 0.38 | 0.54 |
| - | mlKNN | 0.44 | 0.55 | 0.68 | 0.71 | 0.25 | 0.54 | 0.53 |
|  |  | 0.60 | 0.61 | 0.68 | 0.81 | 0.31 | 0.69 | 0.62 |
| idf | mlKNN | 0.55 | 0.48 | 0.58 | 0.49 | 0.21 | 0.57 | 0.48 |
|  |  | 0.66 | 0.54 | 0.58 | 0.55 | 0.27 | 0.73 | 0.55 |

Performance of OWSum and mlKNN (optimized k=1) regarding the prediction of the descriptors ‘floral’, ‘medicinal’, ‘woody, resinous’, ‘sickening’, ‘fruity, other than citrus’ and ‘perfumery’ using five-fold cross-validation. One-versus-rest MCC values are provided per class as well as averaged results over all classes. The first line shows underestimated (lower bound), the second line overestimated (upper bound) values per variant. See the Method Section in the main manuscript for a detailed explanation of these metrics.

**Figure S1. ROC curves per odor for tf-idf-weighted OWSum with idf feature selection.** Five-fold cross-validation was applied to the dataset. The green and yellow lines depict the ROC curve for one-versus-rest for one of those train-test-splits. Green lines: overestimated ROC curves (upper bound), yellow lines: underestimated ROC curves (lower bound). See the Method Section in the main manuscript for a detailed explanation of these metrics.

**
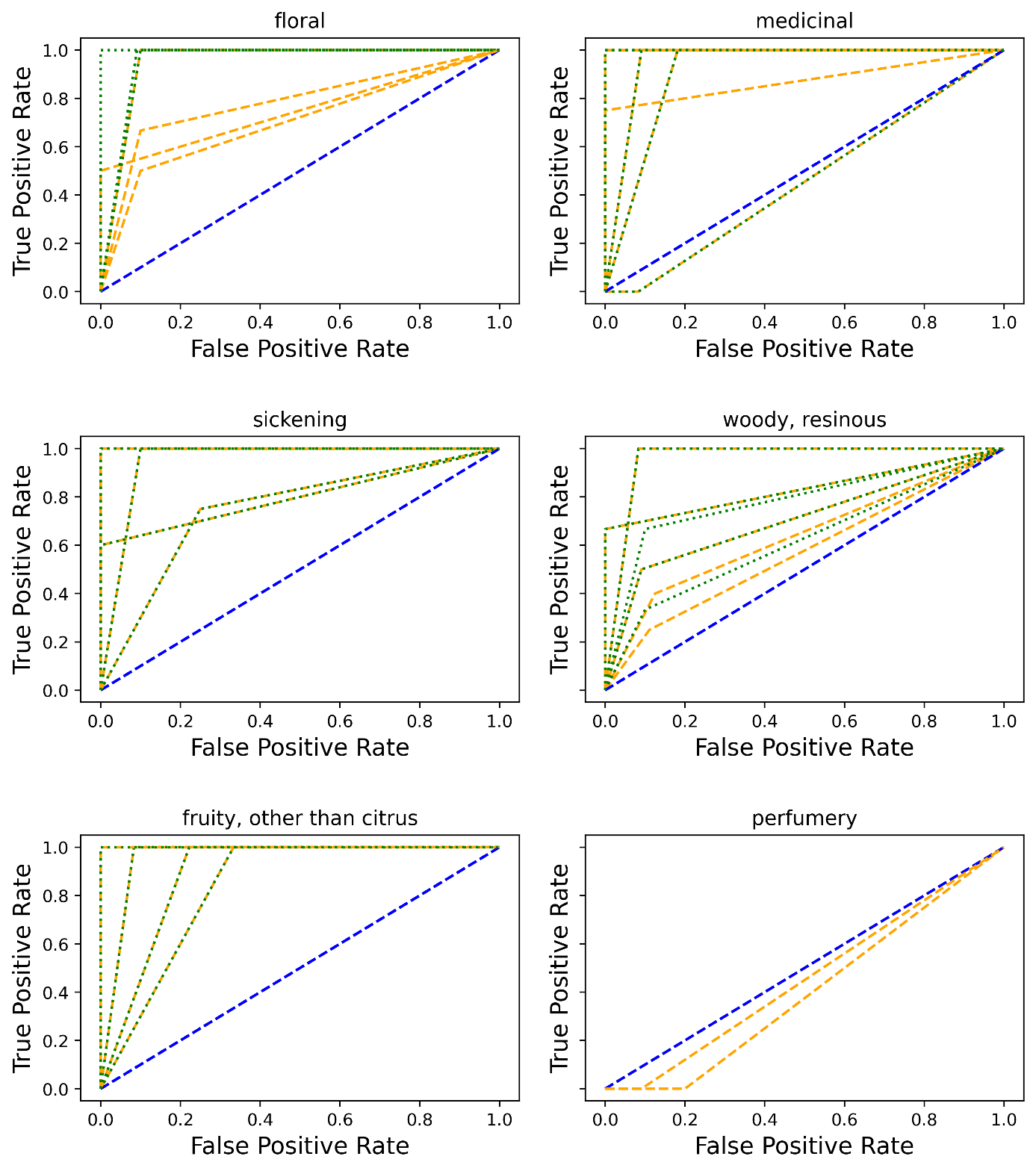
**

**Figure S2. Database characteristics.** We show the number of extracted features for each descriptor in the upper graph. In total 103,643 features and 103,093 features with an idf value higher than zero were extracted. In the bottom graph, we show the number of molecules smelling like a specific descriptor.


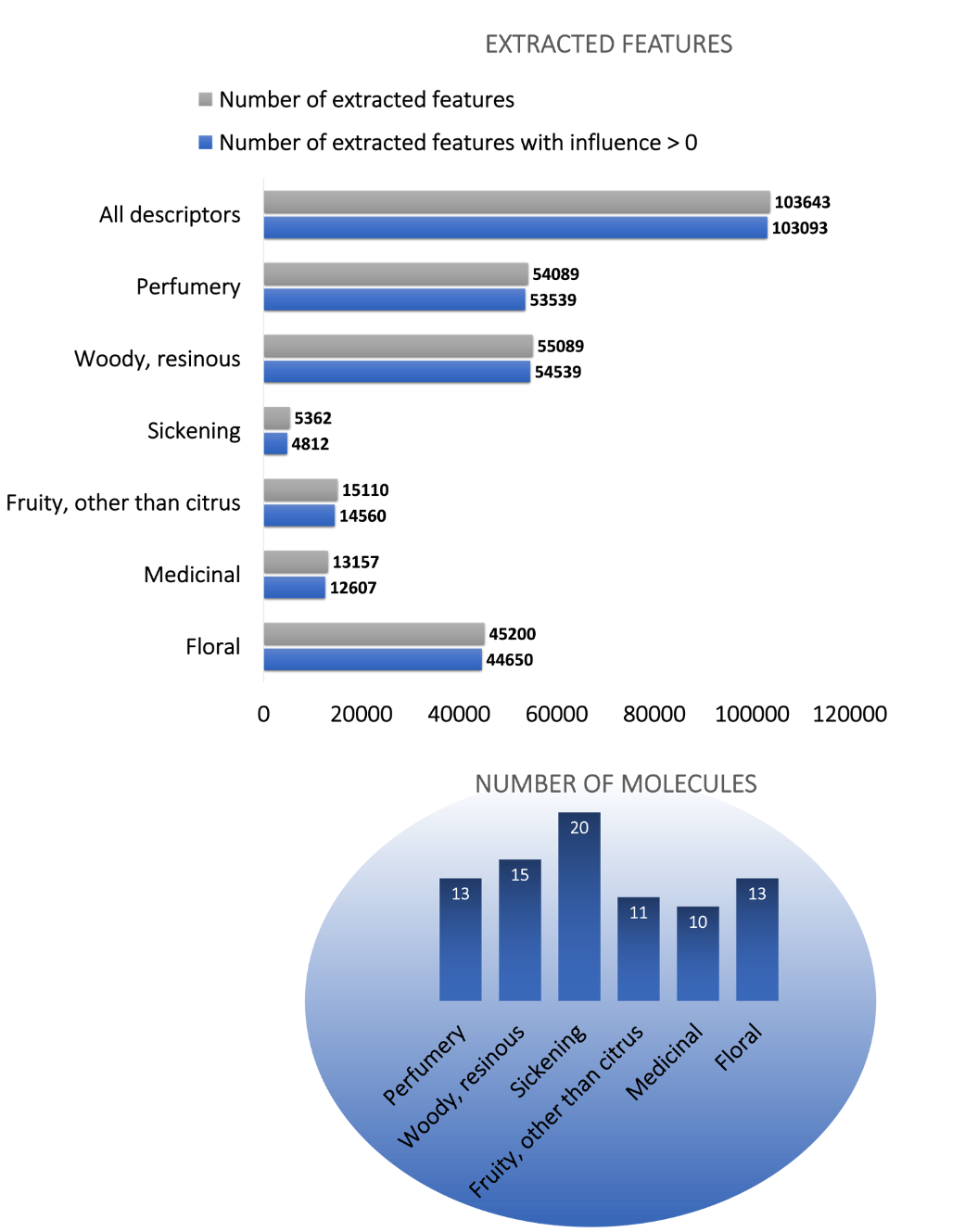


**Figure S3: Example for the calculation of the descriptor overlap.** 21 molecules smell like ‘woody, resinous’ and/or ‘medicinal’. 11 molecules of them only smell like ‘woody, resinous’, 6 only like ‘medicinal’. 4 smell like both descriptors. The resulting descriptor overlap between these two descriptors is 0.40.


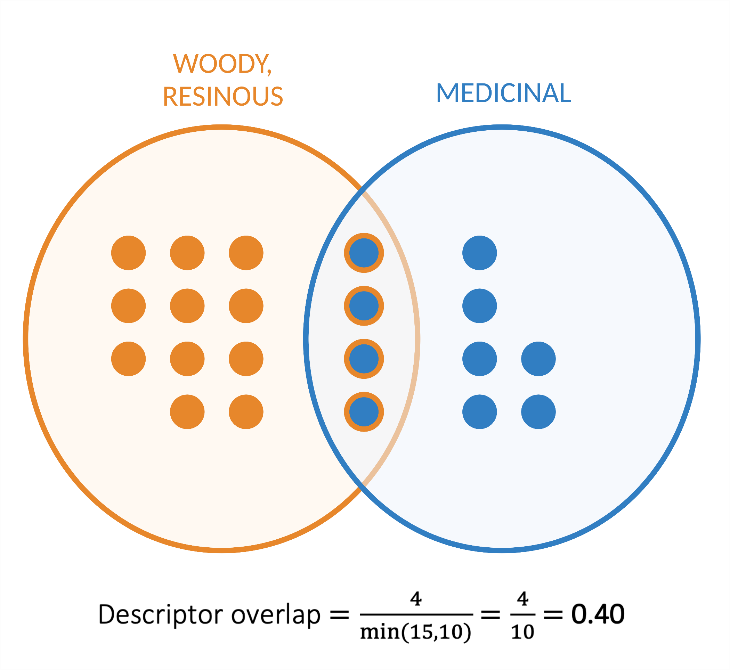

Supplement: Supplementary file 1 — Additional file 1: Table S1. ROC AUC values per odor. Table S2. MCC values per odor. Figure S1. ROC Curves for tf-idf-weighted OWSum with idf-feature selection per odor. Figure S2. Additional image with descriptive information of our database and extracted features. Figure S3. Example for the calculation of the descriptor overlap [file 13321_2023_722_MOESM1_ESM.docx]
